# Supplementary material for: Viral delivery of tissue nonspecific alkaline phosphatase diminishes craniosynostosis in one of two FGFR2C342Y/+ mouse models of Crouzon syndrome
Source: PLoS One. 2020 May 29;15(5):e0234073. doi: 10.1371/journal.pone.0234073 (PMC7259715; doi:10.1371/journal.pone.0234073)
Supplement: Attachment — Submitted filename: 122719 response to critiques.pdf [file pone.0234073.s001.pdf]

Response to editor and reviewer:

We would first like to thank the reviewers for their thorough review and thoughtful critiques. In response to this valuable feedback we have 1) rewritten the manuscript to provide better rationale and background for the study, 2) increased the number of mice to increase power and achieve significance of results, 3) provided additional information on the utilized lentivirus and additional data on serum liver enzyme levels to document and discuss potential influence of the viral vector on results. We believe that the manuscript is significantly improved as a result of these changes. We hope that the editor and reviewers agree that the improved manuscript is now worthy of publication.

Editor's comments:
We note that you have included the phrase "data not shown" in your manuscript. Unfortunately, this does not meet our data sharing requirements. PLOS does not permit references to inaccessible data. We require that authors provide all relevant data within the paper.

This data (evidence of neonatal suture fusion in C57BL/6 Crouzon mice) is now provided as Fig. 1 of the manuscript.

Reviewers' comments:

Reviewer #1:
A major criticism of the manuscript is that there appears to be significant overlap in methods and outcomes when compared to the author's prior publication entitled "The Effects of Tissue-Nonspecific Alkaline Phosphatase Gene Therapy on Craniosynostosis and Craniofacial Morphology in the FGFR2C342Y/+ Mouse Model of Crouzon Craniosynostosis" originally published in 2015. Relatively minor differences in study design are insufficient to justify publication of the present manuscript.

The previously published manuscript utilized archival lots of the virus that were generously provided by a collaborator. The lots had highly variable titers, such that in the end we were only able to investigate a small number of mice. Results were promising though, such that it seemed worthwhile pursuing on a greater scale with better virus. In this study we have utilized freshly prepared virus as well as a greater number of animals. For this revision, we increased the number of mice and performed a closer analysis of craniosynostosis. We are now able to state that injection of the virus into neonatal Crouzon mice does diminish the severity of craniosynostosis and the incidence of malocclusion; though this is dependent upon genetic background of the mice.

Other major criticisms: insufficient detail is provided when describing the lentiviral vector used in the study. Both the viral background and pseudotype should be specified. The authors use a vehicle only control when the appropriate control would include equivalent dosing with a control vector that lacks the TSAP transgene. The authors make no attempt to evaluate the distribution or efficiency of gene transfer.

In response to this valid critique, we now include a more complete description of the virus, including envelop and vector, in the methods sections for TNAP lentivirus. We also include

references that previously established biodistribution of the virus and longevity of efficacy for increasing serum AP levels and health of TNAP deficient mice in a previous study in which the same virus was used to rescue bone abnormalities in the TNAP/Akp2/Alpl knockout mouse model of hypophosphasia.

This raises major concern. The bulk of systemically administered lentiviral particles are filtered by the liver. The viral pseudotypes typically used in research are known to cause damage to the liver. Liver damage, in turn, results in elevated expression of alkaline phosphatase. Thus, it becomes difficult to conclude whether the elevated AP activity observed by the authors is a result of gene transfer or is merely the result of liver damage. A simultaneous evaluation of serum AST and ALT levels (liver aminotransferase) might provide an appropriate control for making this distinction.

In response to this critique, we performed analyses for ALT and AST levels in serum from the mice (Fig. 6). We found that ALT levels were lower while AST levels were higher in Crouzon treated than Crouzon control mice. We then performed studies to determine if serum AP levels correlated with serum ALT and/or AST levels in the treated mice. No correlation was found between serum AP and serum AST levels in the treated mice. A significant reverse correlation was found between serum AP and serum ALT levels in the treated mice. While these results suggest that injection with the lentivirus did alter liver enzyme activity, lack of positive correlations between serum AP levels with serum AST or ALT levels suggests that the increase in serum AP levels seen in the treated mice was likely due to lentiviral expression of TNAP and not due to liver toxicity. It is also worth noting that serum ALT and AST levels did not correlate with body weight, suggesting that any liver toxicity was minor and not overall detrimental to the treated mice. Importantly, we now also include text referring to the method of delivery and limitations of that method in the discussion section of the manuscript (lines 396-408).

Reviewer #2: The manuscript "Tissue nonspecific alkaline phosphatase improves bone quality but does not alleviate craniosynostosis in the FGFR2C342Y/+ mouse model of Crouzon syndrome" describes a study that aims to determine if delivery of TNAP prevents or diminishes FGFR2C342Y driven craniosynostosis. While this is an interesting and worthwhile investigation seeking to identify a pharmacological treatment for craniosynostosis, there are several issues that if addressed could vastly improve the manuscript. The organization overall of the introduction and discussion is lacking. Enough background should be provided in the introduction to understand the rational behind the investigation. The discussion should provide context for the results, in this case discussion of whether or not this intervention is effective. Addition of an assessment of timing of intervention to the discussion may be appropriate as two models were used in the investigation.

In response to this critique, the manuscript has been re-written to provide more background and rationale in the introduction section, with the discussion section more limited to discussing efficacy of the treatment, including timing of intervention.

Major Revisions:
1) It is not clear in the manuscript why two mouse models were used. The authors indicate a difference in severity between the models only in the methods. This fact is not addressed in the introduction, or discussion

Two mouse models were used to test efficacy of the treatment because one model is of neonatal onset and more severe craniosynostosis (C57BL/6 strain) and one model is of postnatal onset and more moderate craniosynostosis (BALB/c strain). We anticipated better rescue in the later onset model of craniosynostosis based upon the fact that treatment would be initiated weeks prior to onset of craniosynostosis in this model. We were surprised to find better rescue in the neonatal onset model of craniosynostosis. Differences between the two models are now described in introduction section. Discussion of differences in treatment efficacy for the two models is also now included in the discussion section.

2) It is not clear why the authors are investigating the cranial base. More background into the areas of interest investigated would be helpful.

That skull growth involves both intramembranous growth of the cranial vault bones and endochondral growth of the cranial base bones is now described in the introduction section. In addition, the fact that craniosynostosis can occur in combination with cranial base growth defects and is present in Crouzon craniosynostosis is also now stated in the introduction section. Cranial base growth defects and craniosynostosis lead to midface retrusion and class III malocclusion (lower front teeth anterior to upper front teeth).

3) Is it possible that the C57BL/6 model is under powered? Was there an assessment for how many individuals per group would be necessary for an appropriately powered analysis?

This critique of the manuscript is of great importance and worth addressing. In response, we increased numbers of C57BL/6 mice and can now state that delivery of recombinant TNAP diminishes severity of craniosynostosis and the incidence of class III malocclusion in the C57BL/6 FGFR2$^{C342Y/+}$ mouse model of Crouzon syndrome. Power analyses on the BALB/c FGFR2$^{C342Y/+}$ craniosynostosis and malocclusion data indicated a need for 1000’s and ~100 mice respectively to achieve a power of 80, so we did not pursue an attempt to achieve power for the BALB/c FGFR2$^{C342Y/+}$ mouse.

4) In the abstract, mention in made methodologically that two mouse models are used while results of only one model are indicated within the abstract.

The last paragraph of the introduction now describes the two mouse models and states why both were used (to see if efficacy was dependent upon timing of coronal suture fusion/craniosynostosis).

5) The first paragraph of the discussion should be in the introduction. This paragraph indicates the rational for conducting the study and establishes the relationship between Crouzon syndrome, FGF signaling and TNAP. Alternatively, the introduction could be expanded to provide this background which could then be again referenced in the discussion with relation to the results of the investigation.

We agree that the previous manuscript version did not provide adequate background and rationale for the study. As suggested by this reviewer, the first paragraph of the discussion is

moved to the introduction in this resubmission.

6) Throughout the paper mice/ groups should be referred to by genotype and treatment, or the currently used shorthand of BALB/c Crouzon etc. should be defined in the Animal Procedures section where the n of each group is defined.

The term “Crouzon” has been replaced with the genotype $FGFR2^{C342Y/+}$ throughout the manuscript.

7) Though there was an indication in the statistical methods that there was an analysis by sex (incorrectly identified as gender), no results or mention of this analysis was indicated. Was the analysis carried out? What were the results?

Results from previous studies and this study demonstrate no sex difference for craniosynostosis in $FGFR2^{C342Y/+}$ mice, so sexes were combined for analyses in this manuscript. This is now stated in the 1st line of the statistics section (line 184-185).

Please note that the statistics section in the methods section (lines 184-192) is different than that which was in the previous submission because we re-analyzed the data after incorporating more mice, to ensure validity of our results.

8) The figure legend for figure 1 does not match the displayed figure, and B cannot be both an axial view and a later view.

Figures 1 and 2 are now combined as a single figure and the figure legend has been corrected (Figure 3).

9) The figure legend for figure 2 does not match the displayed figure, and again B cannot be both an axial and lateral view.

Figures 1 and 2 are now combined as a single figure and the figure legend has been corrected (Figure 3).

10) The figure legend for figure 3 does not indicate the difference between panel A and B.

This figure has been revised to only include coronal suture fusion (Fig. 4), to include a more comprehensive analysis of coronal suture changes. No significant changes were found upon treatment for either the SOS or ISS synchondroses, so these results are summarized within the text.

Minor Revisions:
1) Introduction line 49 “Craniosynostosis occurs in association with…” Please reword this sentence. Currently it implies that all cases of craniosynostosis are the result of Fgfr2 mutations and that is not the CASE.

This sentence has been revised. (lines 51-53)

2) Introduction line 52, “FGF” should be spelled out and parenthetically abbreviated

FGFR2 is now spelled out on line 51.

3) Methods line 112, micro-computed tomography is hyphenated. It occurs elsewhere in the manuscript without the hyphen. Please be consistent.

Hyphens have been removed, such that micro computed tomography is spelled consistently throughout the manuscript.

4) Methods line 122, micro CT is abbreviated though it is not previously defined. Please be consistent.

As stated above, hyphens have been removed, such that micro computed tomography is spelled consistently throughout the manuscript.

5) Methods line 120-122. This sentence appears to have an extra word in it… Perhaps delete dissected, or skulls.

This sentence has been corrected (now lines 153-155).

6) Methods line 128, ISS and SOS have previously been defined and do not need to be defined again in this paragraph.

The definitions have been removed from this sentence (now line 165).

7) When reporting statistical values, it is customary to include a digit before the decimal point (>0.0001).

Statistical values now include a digit before the decimal point.

8) Methods line 148, Please change gender to sex. Mice have a biological sex. Gender is a term used with reference to social and cultural differences rather than biological ones.

Sex has been used instead of gender (line 184).

9) Methods line 150, delete also

This sentence was deleted.

10) Methods line 152, Change Fishers to Fisher’s

This typo has been corrected (line 192).

11) Results line 156, Alkaline phosphatase has already been abbreviated, use the abbreviation

The abbreviation, AP, is now used (line 195).

12) Results line 162, Delete as expected.

“as expected” was removed from this sentence (line 202).

13) Table 4, Please be consistent when referring to treatment, either always as vehicle, or always as PBS.

“PBS” has been replaced with “vehicle” in Table 4 and “vehicle” is used throughout the manuscript.

14) Discussion line 276, HPP has not previously been abbreviated or identified.

HPP is now spelled out before use of abbreviation (line 350).

15) Discussion line 287, Delete

The phrase “to some extent” was deleted from this sentence (line 390).
