# Supplementary material for: Viral delivery of tissue nonspecific alkaline phosphatase diminishes craniosynostosis in one of two FGFR2C342Y/+ mouse models of Crouzon syndrome
Source: PLoS One. 2020 May 29;15(5):e0234073. doi: 10.1371/journal.pone.0234073 (PMC7259715; doi:10.1371/journal.pone.0234073)
Supplement: Attachment — Submitted filename: Response to Reviewers.docx [file pone.0234073.s002.docx]

**Response to Journal:**

1. Please ensure that your manuscript meets PLOS ONE's style requirements, including those for file naming.

References are now in PLOS ONE style.

2. We note that you have stated that you will provide repository information for your data at acceptance. Should your manuscript be accepted for publication, we will hold it until you provide the relevant accession numbers or DOIs necessary to access your data. If you wish to make changes to your Data Availability statement, please describe these changes in your cover letter and we will update your Data Availability statement to reflect the information you provide.

Online data repository information is now provided with the submission.

3. Your ethics statement must appear in the Methods section of your manuscript. If your ethics statement is written in any section besides the Methods, please move it to the Methods section and delete it from any other section. Please also ensure that your ethics statement is included in your manuscript, as the ethics section of your online submission will not be published alongside your manuscript.

Animal use ethics statement including prior approval by university committee, animal protocol number and type of euthanasia performed is now included in the methods section.

**Response to Reviewers:**

We would like to thank the reviewers again for their thorough manuscript review and thoughtful critiques. We appreciate that the reviewers agreed that we: 1) provide a technically sound manuscript in which the data support the conclusions, 2) performed an appropriate and rigorous statistical analysis, 3) made all data underlying the findings in the manuscript fully available and 4) presented the manuscript in an intelligible fashion written in standard English.

We agree with and have made relevant changes in response to both Reviewer #1 and Reviewer #2 critiques and suggestions. The only exception to this is our inability to provide histologic data at this time (due to COVID-19 based university research activity ramp down). We were fully prepared to provide histology at final time points until this ramp down. In an attempt to provide some additional data given the lack of histology, in this revision we have an additional figure showing images of dissected skulls of the mice (Fig. 4). We hope that the editor and reviewers agree that this manuscript submission is worthy of publication.

**Reviewer #1:** The manuscript “Tissue nonspecific alkaline phosphatase improves bone quality but does not alleviate craniosynostosis in the FGFR2C342Y/+ mouse model of Crouzon syndrome” has been revised well. In particular, the changes made to the introduction and discussion have improved the manuscript significantly. The study that aims to determine if delivery of TNAP prevents or diminishes FGFR2C342Y driven craniosynostosis. This is an interesting and worthwhile investigation seeking to identify a pharmacological treatment for craniosynostosis. Though the authors have addressed the major concerns from the previous review, a number of minor issues still need to be addressed.

**Minor Revisions:**
1. Abstract Line 33: The use of the word improved is vague. As you have not defined the quality/ described normal/ unaffected bone, improved is confusing. Consider changing this language to be directional, as in “increased bone volume as compared to control.”

We agree that “improved” is vague. The word has been replaced by “increased” and “as compared to controls” has been added in the abstract.

2. Introduction line 65: “… we found statistical differences” – What does this mean? Perhaps significant would be a better word in place of statistical?

The word “statistical” has been replaced with “significant” in this sentence.

3. Introduction lines 74-76: Consider rephrasing. The way that this is written indicates one model of Crouzon syndrome, and though only one genetic target was tested here there are in fact two different models of the human disease being studied. Please rephrase the second half of this sentence “ a model of Crouzon craniosynostosis syndrome”

This phrase has been replaced with, “in BALB/c FGFR2^C342Y/+^ mice, a model of less severe Crouzon craniosynostosis syndrome and/or in C67BL/6 FGFR2^C342Y/+^ mice, a model of more severe Crouzon craniosynostosis syndrome” in the introduction section to the manuscript (lines 75-77).

4. Methods section Micro Computed Tomography, Line 149: Please be consistent with referring to Micro Computed Tomography. Either always write it out or abbreviate it at the first instance. It is in different forms (written out, micro CT, micro-CT) throughout the methods, figure legends, and results.

To eliminate inconsistencies with regard to terminology for micro computed tomography, the methods section is now titled: Micro Computed Tomography (micro CT) and all references within the text of the manuscript have been changed to “micro CT).

5. Methods section Statistics, Line 186, 187: There are extra words in this sentence. Please revise carefully.

We apologize for this confusing sentence and thank the reviewer for pointing this out. The sentence has been revised with elimination of extra words, which should clarify interpretation of text (lines 187-189).

6. Results Table 1, Lines 204-205: Please be consistent with capitalization in the table title. If measurements is capitalized, then vehicle, treatment, and mice should also be capitalized. Also, be consistent with table format. Have the heading bolded all the time or none of the time.

Thank you for catching these errors. All tables now have bolded headings. Capitalization of sentences should also now be consistent.

7. Results Table 2, Lines 231-232: Please be consistent with capitalization in the table title. Also, be consistent with table format. Have the heading bolded all the time or none of the time.

All tables now have bolded headings. Capitalization of sentences should also now be consistent.

8. Results, Line 249: This seems to be the first instance of the term “wild type” please use the gene instead as I believe you mean unaffected and not a true wild-type.

For improved clarity, “wild type” is now referred to as FGFR2^+/+^ throughout the manuscript.

9. Results Table 3, Lines 272-273: Please be consistent with capitalization in the table title. Also, be consistent with table format. Have the heading bolded all the time or none of the time.

All tables now have bolded headings. Capitalization of sentences should also now be consistent.

10. Results, Line 296: The term “wild type” is used again and I again believe you mean unaffected, which would make using the gene notation more appropriate.

“wild type” is now referred to as FGFR2^+/+^ throughout the manuscript.

11. Results Table 4, Lines 302-303: Please be consistent with capitalization in the table title. Also, be consistent with table format. Have the heading bolded all the time or none of the time.

All tables now have bolded headings. Capitalization of sentences should also now be consistent.

12. Results Line 324-327: Please revise this sentence to the following as data is always plural, and the sentence in currently in multiple tenses: “Together, these data show that the effect of lentiviral TNAP treatment decreased ALT and increased AST enzymes in serum, but had not effect on body weight of the mice and was not likely the cause of the observed increase in serum AP levels in treated mice.”

The final sentence of results sections on ALT/AST levels (lines 346-349) has been changed to the reviewer suggested sentence (lines 395-398).

13. Discussion Line 330: The use of the word improved here is also vague. Consider changing this language as suggested above.

Thank for this reminder to avoid ambiguous terminology. The word “improved” has been changed to “increased” here. Use of the word “improved” has also been eliminated from the manuscript text (line 352).

14. Discussion Line 351: delete “does”

This word has been deleted (line 452).

15. Discussion Line 352: As data is always a plural (datum is singular) supports should be changes to support. Please check for agreement of this throughout your discussion, Lines 356, 368,369 need to be revised.

The word “supports” has been changed to “support” here. Grammar has also been corrected to consistently refer to data as plural in the rest of the discussion text.

16: Discussion line 369: is there a functional difference between point fusions across the suture and complete obliteration? Perhaps this could factor into the discussion to some degree as a means of better situating this study within a clinical context in the treatment of craniosynostosis.

This is an excellent point. Discussion of surgical risk for correction of point fusions vs. suture obliteration are now included at the end of the discussion section, including an additional reference (lines 516-522).

17. Figure Legends Figure 1, Line 571: As you have indicated that the fusion of the coronal suture occurs early (pre-natally) in the C57BL/6 model please indicate that the images here are from post-natal day 3 mice. Also, within Figure 1 the notation should be consistent. Either always CZ or always Crouzon, and always C57BL6 or C57Bl6. Please check that you are consistent with these notations throughout the manuscript, tables, legends, and figures.

The phrase “day three” has been replaced with “post-natal day 3” mice in the legend for Figure 1.

C57BL/6 is now used consistently throughout text, tables and figure legends.

18. Figure Legends Figure 4, Line 592: Please be consistent throughout the manuscript, particularly within figures and legends. Either use Crouzon or CZ, not both.

Figure legends for figures 4 and 5 now refer to Cz as opposed to Crouzon, to be consistent with other figure legends. The figures have also been changed so as to refer to Cz, not Crouzon.

19. Figure 3: what does the notation no tx vs. tx stand for? This is not clear in the legend.

The legend for figure 3 is now labeled “control” as opposed to no tx. The legend also now includes reference to the fact that “tx” refers to lentiviral TNAP delivery and control refers to no lentiviral delivery of TNAP.

20. Figure 4. Please revise this figure to have on legend that includes all of the colors and display if possible all colors on each graph. Also, please use proper and consistent capitalization on titles throughout this figure and all other figures.

Figure 5 (previously figure 4) is revised to include all colors for all grades of suture fusion. Revision of figure title also now has consistent title capitalization.

21. Figure 5: Why are there not titles on these graphs?

Figure 6 (previously figure 5) now has titles above graphs and on the vertical axis.

22. Figure 6: Please indicate on the graphs and or in the legend which strain was used for these experiments.

The strain is now indicated in the legend for figure 7 (previously figure 6).

**Reviewer #2:** Overall this is an interesting study, but it feels unfinished, even though the work presented here is a continuation -with significant overlap- from previous work. The study can easily be improved by the addition of histology data of the coronal suture for example. If the treatment with TNAP does indeed delay or reverse the process of synostosis this could have been shown by a longitudinal study of the suture, analysing the morphology of the suture and the histology of the sutural mesenchyme.

We agree that it would have been ideal to do analyses at earlier time points of the treatment to determine if treatment delayed or reversed fusion. Had this reviewer suggestion been provided in the critique of the 1^st^ manuscript submission, we would have euthanized mice at internal time points to perform histology while we were generating additional control and treated mice to obtain power for the 2^nd^ submission. At this point, we hope that expanded discussion of this limitation is adequate for publication (lines 427-428).

Additionally, we had planned to provide histologic sections of animals from groups at the final time points for this submission. Unfortunately, in this time of COVID-19, we have been unable to pursue these studies in a timely manner to meet the resubmission deadline. We are fully prepared to provide histology of bone and suture, but will not be able to do so until our university again allows for research activity. If histology is deemed necessary, we will proceed but cannot now say when that can be completed.

In an attempt to address this critique under current circumstances in which we cannot pursue histology, this revision includes an additional figure (Figure 4) showing the coronal suture in dissected C57BL/6 mouse skulls. We recognize that this is not the same as histology but, given the poor bone mineralization and therefore difficulty visualizing the micro CT images in Figure 3, we thought inclusion of Figure 4 would help with visualization of the rescued coronal suture in a TNAP treated mouse.

Material and methods – Linear measurements: I don’t understand why the authors decided to perform skull measurements using a caliper when they have microCT scans available. Quantitative data using microCT scans is far superior. It would be helpful if the authors could explain why they have decided to not do this.

We did not include micro CT based linear methods because we have done both micro CT based linear measurements plus digital caliper based measurements for numerous mouse models and find that measurements from the two methods yield similar results. So now we have taken to commonly providing only digital caliper based measurements because this method produces reproducible, high quality, quantifiable data that can provide phenotype information efficiently and prior to micro CT.

Material and methods – Statistics: While the authors mention a previous study that found no differences between male and female Fgfr2-C342Y mice regarding the craniosynostosis phenotype, it is currently considered bad practice to use a mixed sex cohort, especially when testing a pharmacological intervention. I recommend the authors change this in the future.

Thank you for this suggestion. We will proceed in the future by not using a mixed sex cohort.

Discussion: It would be interesting if the authors could comment on the feasibility of administering TNAP in utero.

Discussion of in utero TNAP delivery is now included (lines 432-444).

The term perinatal is not used correctly. It refers to the period shortly before to shortly after birth. In the

Abstract (line 35) perinatal is used where it should be postnatal. Also, in line 67, the addition of the term perinatal to prenatal and postnatal is superfluous.

Perinatal has been replaced with the term postnatal in the abstract (line 35) and perinatal has been removed from the introduction section describing craniosynostosis onset (line 67).

In Figure 3, it would be helpful if the authors commented on if the images are representative for their cohorts.

Figure 3 legend now includes reference to the fact that mice in these images are representative of their cohorts.

In addition, introduction to the craniofacial linear measurement data now includes the phrase, “Consistent with images shown in Fig. 3” (line 243).

The quality of the images in D and F (arguably the most interesting) is poor due to a likely issue with contrast. As a result the coronal sutures -or what’s left of them- are impossible to see.

It is true that isosurface micro CT images of the C57BL/6 FGFR2^C342Y/+^ mice in figure 3 (D,F) are difficult to interpret for coronal suture fusion. This is because the cranial bones of these mice are so poorly mineralized that you can see through them to the cranial base, such that the cranial base and cranial vault appear superimposed when the image is taken at a bone limited threshold. To address this concern, we now also include micro CT isosurface images of the C57BL/6 FGFR2^C342Y/+^ mice at a lower threshold that includes both bone and soft tissue. Please note that we did not include comments on coronal suture fusion assessment with these images, because it is not appropriate to do so. These images provide overall skull morphology.

In Table 3, the # referring to the non-significant differences is missing.

Thank you for noting this. “^#^ p value < 0.01 between treatment groups” is now written under Table 3.
