# Supplementary material for: Viral delivery of tissue nonspecific alkaline phosphatase diminishes craniosynostosis in one of two FGFR2C342Y/+ mouse models of Crouzon syndrome
Source: PLoS One. 2020 May 29;15(5):e0234073. doi: 10.1371/journal.pone.0234073 (PMC7259715; doi:10.1371/journal.pone.0234073)
Supplement: Attachment — Submitted filename: Response to Reviewers.docx [file pone.0234073.s003.docx]

**PONE-D-20-01614R1**
Viral delivery of tissue nonspecific alkaline phosphatase diminishes craniosynostosis in one of two FGFR2C342Y/+ mouse models of Crouzon syndrome.
PLOS ONE

Dear Dr Hatch,

Thank you for submitting your manuscript to PLOS ONE. After careful consideration, we feel that it has merit but does not fully meet PLOS ONE’s publication criteria as it currently stands. Therefore, we invite you to submit a revised version of the manuscript that addresses the points raised during the review process.

There are a couple of outstanding point with the figures that should be addressed prior to final approval.

We would appreciate receiving your revised manuscript by Jun 26 2020 11:59PM. When you are ready to submit your revision, log on to <https://www.editorialmanager.com/pone/> and select the 'Submissions Needing Revision' folder to locate your manuscript file.

If you would like to make changes to your financial disclosure, please include your updated statement in your cover letter.

To enhance the reproducibility of your results, we recommend that if applicable you deposit your laboratory protocols in [protocols.io](http://protocols.io), where a protocol can be assigned its own identifier (DOI) such that it can be cited independently in the future. For instructions see: <http://journals.plos.org/plosone/s/submission-guidelines#loc-laboratory-protocols>

Please include the following items when submitting your revised manuscript:

- A rebuttal letter that responds to each point raised by the academic editor and reviewer(s). This letter should be uploaded as separate file and labeled 'Response to Reviewers'.
- A marked-up copy of your manuscript that highlights changes made to the original version. This file should be uploaded as separate file and labeled 'Revised Manuscript with Track Changes'.
- An unmarked version of your revised paper without tracked changes. This file should be uploaded as separate file and labeled 'Manuscript'.

Please note while forming your response, if your article is accepted, you may have the opportunity to make the peer review history publicly available. The record will include editor decision letters (with reviews) and your responses to reviewer comments. If eligible, we will contact you to opt in or out.

We look forward to receiving your revised manuscript.

Kind regards,

JJ Cray Jr., Ph.D.
Academic Editor
PLOS ONE

**Reviewers' comments:**

**Comments to the Author**

1. If the authors have adequately addressed your comments raised in a previous round of review and you feel that this manuscript is now acceptable for publication, you may indicate that here to bypass the “Comments to the Author” section, enter your conflict of interest statement in the “Confidential to Editor” section, and submit your "Accept" recommendation.

Reviewer #1: All comments have been addressed
Reviewer #2: (No Response)

2. Is the manuscript technically sound, and do the data support the conclusions?

The manuscript must describe a technically sound piece of scientific research with data that supports the conclusions. Experiments must have been conducted rigorously, with appropriate controls, replication, and sample sizes. The conclusions must be drawn appropriately based on the data presented.

Reviewer #1: Yes
Reviewer #2: Yes

3. Has the statistical analysis been performed appropriately and rigorously?

Reviewer #1: Yes
Reviewer #2: Yes

4. Have the authors made all data underlying the findings in their manuscript fully available?

The [PLOS Data policy](http://www.plosone.org/static/policies.action#sharing) requires authors to make all data underlying the findings described in their manuscript fully available without restriction, with rare exception (please refer to the Data Availability Statement in the manuscript PDF file). The data should be provided as part of the manuscript or its supporting information, or deposited to a public repository. For example, in addition to summary statistics, the data points behind means, medians and variance measures should be available. If there are restrictions on publicly sharing data—e.g. participant privacy or use of data from a third party—those must be specified.

Reviewer #1: Yes
Reviewer #2: Yes

5. Is the manuscript presented in an intelligible fashion and written in standard English?

PLOS ONE does not copyedit accepted manuscripts, so the language in submitted articles must be clear, correct, and unambiguous. Any typographical or grammatical errors should be corrected at revision, so please note any specific errors here.

Reviewer #1: Yes
Reviewer #2: Yes

6. Review Comments to the Author

Please use the space provided to explain your answers to the questions above. You may also include additional comments for the author, including concerns about dual publication, research ethics, or publication ethics. (Please upload your review as an attachment if it exceeds 20,000 characters)

**Reviewer #1:** The authors of this manuscript have done a very careful revision. I would like to thank them for their careful attention to consistency of language and their revision of word choice away from vague terms. I have very few minor comments as follow:

Line 138-139: There are extra words in this sentence. I suggest deleting “as the.” This word was deleted.

Line 168-169: Please spell out ICC – ICC is now spelled out as intraclass correlation coefficient.

Table 2: If possible please add the ones place digit for all values (0.12 instead of .12) Thank you for pointing this out. A digit was added at the ones place for all values.

Table 4: If possible please add the ones place digit for all values (0.12 instead of .12). If not possible, please be consistent and never have the ones place digit included when it is 0. Thank you for pointing this out. A digit was added at the ones place for all values.

Line 640: Missing word, revise to “underlying cranial base due to translucent poorly mineralized”… Thank you for catching this. The phrase was revised for this submission.

Figure 4 legend: Please add “Tx indicates delivery of TNAP” to the legend. This figure was removed per Reviewer #2 request.

**Reviewer #2:** The authors have addressed all of the minor and the paper -especially the Discussion- has improved. However, the two main concerns are still left somewhat unresolved.

• Regarding caliper versus microCT measurements of skull dimensions, the authors claim that they “…find that measurements from the two methods yield similar results.” If this is the case I would have expected the authors to either show this as part of this paper or refer to previously published work. Mainly because this is counter-intuitive and because this will be very useful information for colleagues performing similar analyses in other laboratories. However, I will take the authors on their word that this is indeed the case.

We agree with reviewer #2 that there are situations in which a micro CT based morphologic analysis is appropriate, particularly (for example) if there is a need for performing shape analyses that require 3D coordinate data of landmarks, or analyses of specific regions of the skull that cannot be assessed using caliper measurements on dissected skulls. For example, in this manuscript we measured cranial base bones on micro CT images because these measurements cannot be performed well on dissected skulls.

That said, we have found that digital caliper measurements of the overall skull, if done accurately, can relatively quickly convey differences in craniofacial shape, and that data from digital caliper measurements reflects data from micro CT based measurements. We first discovered this working with the *Alpl*^-/-^ mouse model of infantile hypophosphatasia. The craniofacial bones of *Alpl*^-/-^ mice are present but severely under-mineralized, making visualization of some skeletal landmarks difﬁcult on micro computed tomographic images. Therefore, at that time we proceeded with digital caliper measurements using landmarks that included ﬁve standard measurements in use by the Craniofacial Mutant Mouse Resource of the Jackson Laboratory (Bar Harbor, ME). Results demonstrated that *Alpl*^-/-^ mice are acrocephalic (taller) and brachycephalic (wider) relative to anterior-posterior length when compared to *Alpl*^+/+^ mice.^1^ We subsequently performed a more comprehensive morphologic analysis of the same set of mice using micro CT. Results again showed that *Alpl*^-/-^ mice are acrocephalic (taller) and brachycephalic (wider) relative to anterior-posterior length when compared to *Alpl*^+/+^ mice, and that only those *Alpl*^-/-^ mice with a severe bone hypomineralization defect develop the abnormal craniofacial shape.^2^ We have now performed digital caliper and micro CT based measurements on additional mouse models and find that conclusions of results from both methods are similar. We find that the most important factor in reporting accurate skull morphologic differences is appropriate normalization for mouse/skull size differences (regardless of method used to create craniofacial linear measurements). As primary author, I am extremely confident that use of the digital calipers for linear measurements is appropriate in this context, and that the skull morphology data reported in this manuscript accurately reflects what is seen in the mice.

1.	Liu J, Nam HK, Campbell C, Gasque KC, Millan JL, Hatch NE. Tissue-nonspecific alkaline phosphatase deficiency causes abnormal craniofacial bone development in the Alpl(-/-) mouse model of infantile hypophosphatasia. *Bone* 2014;67:81-94.

2.	Durussel J, Liu J, Campbell C, Nam HK, Hatch NE. Bone mineralization-dependent craniosynostosis and craniofacial shape abnormalities in the mouse model of infantile hypophosphatasia. *Dev Dyn* 2016;245:175-182.

• The paper’s main finding is that “Viral delivery of tissue nonspecific alkaline phosphatase diminishes craniosynostosis…” (from the title). This is supported by data in Figure 3 and 5, which highlights the effects of TNAP treatment on C57BL/6 Crouzon mice. I hope the authors agree that scoring the patency of the suture by looking at the external surface only tells part of the story, hence my request of a histological analysis of the underlying suture pathology.

The new data in Figure 4 does* not add anything to support the effect of TNAP on the coronal suture and I suggest the authors remove this figure.

We agree that Figure 4 does not add additional supportive data. We provided it because the images were available during this time in which we cannot return to the laboratory to generate additional data. For this revision, Figure 4 was removed from the manuscript.

The new images in Figure 3 are a useful addition, but I would like to see the same image (using the soft tissue threshold) for the WT to make it possible to compare the ‘rescued’ C57BL/6 suture with the WT one.

In response to this request, an image of a representative WT C57BL/6 mouse using the soft tissue threshold is now included in Figure 3.

Under the circumstances I am prepared to accept the paper without the histological data (partly because of the improved Figure 3), but I am very much looking forward to a follow-up study that analyses the impact of TNAP treatment on the coronal suture bone and mesenchyme at the cellular and molecular level. This study represent some extremely interesting findings and it deserves a more thorough investigation.

This author wants to thank Reviewer #2 for their appreciation both of the significance of results shown here, and of how the current laboratory closures are negatively impacting our ability to generate additional data. We are very interested in understanding how TNAP and FGF signaling interact to control craniofacial development and will most certainly report more mechanistic cell and tissue data in future manuscripts.

Minor points
• Figure 1 Please include an image of the BALB/c strain at the same time point if available. I didn’t spot this in the previous submission, but it would help to highlight the difference between the two Crouzon mouse strains here.

In response to this request, Figure 1 now includes images of the BALB/c strain at the same time point.

• Table 2, line 255 “Measures are reported as normalized to total skull length.” Measures should be measurements.

Thank you for catching this typo. It was corrected for this resubmission.

• Table 3, line 312 “No significant differences between treatment groups were found.” I suspect this should be deleted.

This line was deleted.

*in my opinion (and the Oxford English Dictionary’s) the word data should be treated as a mass noun and thus takes a singular verb.

Controversy regarding use of data as singular or plural has been ongoing for the past century. All verbs for data were previously revised based upon reviewer #1’s critique of a previous resubmission (“Discussion Line 352: As data is always a plural (datum is singular) supports should be changes to support. Please check for agreement of this throughout your discussion, Lines 356, 368,369 need to be revised”).

This author is happy to treat the term data as singular or plural, if the reviewers can agree on which it should be. For now, in this 3^rd^ resubmission, data remains treated as plural, based upon earlier reviewer #1 feedback.

While revising your submission, please upload your figure files to the Preflight Analysis and Conversion Engine (PACE) digital diagnostic tool, <https://pacev2.apexcovantage.com/>. PACE helps ensure that figures meet PLOS requirements. To use PACE, you must first register as a user. Registration is free. Then, login and navigate to the UPLOAD tab, where you will find detailed instructions on how to use the tool. If you encounter any issues or have any questions when using PACE, please email us at [figures@plos.org](mailto:figures@plos.org). Please note that Supporting Information files do not need this step.
